# Silk/Fibroin Microcarriers for Mesenchymal Stem Cell Delivery: Optimization of Cell Seeding by the Design of Experiment

**DOI:** 10.3390/pharmaceutics10040200

**Published:** 2018-10-24

**Authors:** Carlotta Perucca Orfei, Giuseppe Talò, Marco Viganò, Sara Perteghella, Gaia Lugano, Francesca Fabro Fontana, Enrico Ragni, Alessandra Colombini, Paola De Luca, Matteo Moretti, Maria Luisa Torre, Laura de Girolamo

**Affiliations:** 1 IRCCS Istituto Ortopedico Galeazzi, Orthopaedic Biotechnology Lab, Via R. Galeazzi 4, 20161 Milano, Italy; carlotta.perucca@grupposandonato.it (C.P.O.); marco.vigano@grupposandonato.it (M.V.); gaia.lugano@grupposandonato.it (G.L.); enrico.ragni@grupposandonato.it (E.R.); alessandra.colombini@grupposandonato.it (A.C.); deluca.paola@grupposandonato.it (P.D.L.); laura.degirolamo@grupposandonato.it (L.d.G.); 2 IRCCS Istituto Ortopedico Galeazzi, Cells and Tissue Engineering Laboratory, Via R. Galeazzi 4, 20161 Milano, Italy; giuseppe.talo@grupposandonato.it (G.T.); matteo.moretti@grupposandonato.it (M.M.); 3 Department of Drug Sciences, University of Pavia, via T. Taramelli 12, 27100 Pavia, Italy; sara.perteghella@unipv.it (S.P.); ffrancesca.fontana@gmail.com (F.F.F.)

**Keywords:** mesenchymal stem cells, microcarrier, design of experiment, cell delivery, silk fibroin, alginate, one-step clinical procedure

## Abstract

In this methodological paper, lyophilized fibroin-coated alginate microcarriers (LFAMs) proposed as mesenchymal stem cells (MSCs) delivery systems and optimal MSCs seeding conditions for cell adhesion rate and cell arrangement, was defined by a Design of Experiment (DoE) approach. Cells were co-incubated with microcarriers in a bioreactor for different time intervals and conditions: variable stirring speed, dynamic culture intermittent or continuous, and different volumes of cells-LFAMs loaded in the bioreactor. Intermittent dynamic culture resulted as the most determinant parameter; the volume of LFAMs/cells suspension and the speed used for the dynamic culture contributed as well, whereas time was a less influencing parameter. The optimized seeding conditions were: 98 min of incubation time, 12.3 RPM of speed, and 401.5 µL volume of cells-LFAMs suspension cultured with the intermittent dynamic condition. This DoE predicted protocol was then validated on both human Adipose-derived Stem Cells (hASCs) and human Bone Marrow Stem Cells (hBMSCs), revealing a good cell adhesion rate on the surface of the carriers. In conclusion, microcarriers can be used as cell delivery systems at the target site (by injection or arthroscopic technique), to maintain MSCs and their activity at the injured site for regenerative medicine.

## 1. Introduction

The research on stem cell-based therapies is rapidly evolving but, despite the promising results, the translation process from basic research to clinical practice is facing several hurdles in terms of practical and regulatory issues. As demonstrated by recent findings, the therapeutic function of Mesenchymal Stem Cells (MSCs) is not only related to their multi-differentiation potential, but also to their immunomodulatory and trophic activities exerted through the release of a plethora of different molecules with a paracrine function on resident cells [1,2,3,4,5,6,7,8]. Especially in the context of musculoskeletal disorders, therapies involving local administration of MSCs through injection would represent a preferred strategy, thanks to the non-invasive nature of the procedure. 

MSCs-based treatments may include one and two step procedures, using non-expanded or in vitro cultured cells, respectively. While the latter approach allows for the selection of a more homogeneous and standardized cell population [9], it is very expensive and thus hardly affordable. Moreover, it requires extensive in vitro cell manipulation, falling in the field of advanced-therapy medicinal products (ATMPs), and requiring the satisfaction of rigorous regulatory requirements for the translation in clinical practice [10,11]. The minimal manipulation of cells according to the current European Directives for cell therapy allows some of these limitations to be overcome [10,11,12]. Indeed, starting from a minimally invasive harvesting of bone marrow or adipose tissue, it is possible to concentrate the MSC population present in these tissues by commercially-available disposable devices [13]. Since they do not imply substantial cell manipulation and are performed at the point of care, these products are not considered ATMPs and they would reduce the costs and the patients’ discomfort. 

Nowadays, the correct targeting of the injury site represents a main technical concern in the field of MSCs-based therapies, and many studies have been performed in recent years to investigate different MSC-delivery strategies, in the presence or absence of specific carriers [14,15,16,17]. In fact, despite the well-known homing ability of MSCs, allowing them to migrate to the site of injury [18,19], it has been demonstrated that, in the case of systemic administration, only a small number of cells would actually reach the target tissue [18,20,21,22,23]. 

Therefore, the local injection of MSCs in association with biomaterials would improve their presence at the injury site, allowing the maintenance of their physiological status of adherent cells and thus promoting their action [24].

Among medical devices, microcarriers represent a valid tool to enhance the rate of cell delivery at the target site, leading to an improvement of MSCs therapeutic potential [25,26,27,28,29,30,31,32,33,34]. 

Starting from the positive findings of a previous study reporting the use of silk fibroin mats to subcutaneously deliver stromal vascular fraction in the murine model [35], biocompatible silk fibroin-coated alginate microcarriers (FAMs) were developed as a multiparticulate injectable cell-carrier device to deliver expanded or freshly isolated cells [36,37]. Despite providing rapid cell adhesion, the seeding protocol applied in the previous study just allowed for a non-homogeneous arrangement of human adipose-derived stem cells (hASCs) on the surface of the carriers, with formation of cell clusters. 

Therefore, the goal of the present study was to optimize the cell seeding process of MSCs on the surface of Lyophilized FAMs (LFAMs), ameliorating the cell adhesion rate, the cell arrangement on the surface of FAMs, and the whole time of the process while maintaining the cell viability for the duration of the process. A dry product (LFAMs) can be considered as more stable, from a physico-chemical point of view, with respect to a fresh product (FAMs). The water content of FAMs significantly reduced their shelf-life and, for this reason, we performed the lyophilization. 

Given the high number of parameters considered, the principles of Quality by Design (QbD) were applied to this study to improve the efficiency of the investigation. During recent years, the application of the QbD concept has been widely adopted in pharmaceutical research to improve and optimize drug formulations, and to reduce the risks of failure, through the standardization and automatization of the procedures [38]. In this context, the Design of Experiment (DoE) is fundamental to satisfy the QbD principles. DoE employs the statistical principles of randomization, orthogonality, and data distribution to identify the effect of the process variables and the synergistic mechanism between them, using an optimized number of experiments [39]. 

## 2. Materials and Methods

### 2.1. Lyophilized-FAMs Preparation and Characterization

Fibroin-coated alginate microcarriers (FAMs) were fabricated as previously described [36,40,41,42]. Briefly, sodium alginate (1% *w*/*v*, medium viscosity, Sigma-Aldrich, St. Louis, MO, USA) was solubilized in distilled water and then the solution was added dropwise into an aqueous solution containing calcium chloride (Sigma-Aldrich) 100 mM under magnetic stirring using a bead generator (Encapsulator VAR V1, Nisco Engineering AG, Zurich, Switzerland) to obtain alginate microcarriers (AMs). AMs were divided into two aliquots, the first was lyophilized (LAMs) for further analyses (Fourier Transform Infrared FT-IR Spectroscopy) while the second was used for the coating procedure.

*Bombyx mori* cocoons were degummed and silk fibroin fibers were solubilized in phosphoric acid/formic acid (80:20 *v*/*v*) (Sigma-Aldrich) under magnetic stirring at room temperature (RT); the obtained silk fibroin solution was dialyzed against distilled water (membrane cut off 12 kDa, Visking, London, UK) at RT. Alginate microcarriers (AMs) were shaken into fibroin solution (volume ratio alginate microcarriers: fibroin solution 1:2) and then immersed in 96% (*v*/*v*) ethanol (Carlo Erba Reagents, Milan, Italy) to induce silk conformational transition. The procedure was performed three times to assure the homogeneous and complete coating of fibroin-coated alginate microcarriers (FAMs). FAMs were then washed with distilled water and subjected to a freeze-drying process (8 × 10^−1^ mbar, −50 °C for 72 h; Modulyo^®^ Edwards Freeze Dryer, Kingston, NY, USA). Lyophilized fibroin-coated alginate microcarriers (LFAMs) were stored at RT.

Granulometric analysis of LAMs and LFAMs was performed with a laser light scattering granulometer (Beckman Coulter LS230, Miami, FL, USA) equipped with small cell volume (120 mL volume, obscuration 5%). FAMs were suspended in aqueous solution, transferred to the measurement cell and run (5 replicates of 90 s each); while LFAMs were previously rehydrated in distilled water (2 h at 37 °C) and then analyzed. 

FT-IR spectra of LAMs and LFAMs were obtained using a Spectrum One Perkin-Elmer spectrophotometer (Perkin Elmer, Wellesley, MA, USA) equipped with a MIRacle™ ATR device (Pike Technologies, Madison, WI, USA). The IR spectra in transmittance mode were recorded in the spectral region of 650–4000 cm^−1^ with a resolution of 4 cm^−1^. Each experiment was performed in triplicate.

### 2.2. Cell Isolation and Culture in Monolayer

Adipose tissues and bone marrow aspirates were obtained at the Galeazzi Orthopaedic Institute from patients who underwent aesthetic liposuction and hip replacements, respectively. All the procedures involving the use of waste human biological material were carried out according to our Institutional Review Board approval (M-SPER-015-Ver. 2-04.11.2016).

Waste portions of adipose tissue were collected from female and male donors (44 ± 11 years old) who underwent abdominal liposuction. The samples were washed with Phosphate Buffered Saline (PBS) and centrifuged at 1200× *g* for 2 min to remove blood and other contaminants. Human ASCs were collected after enzymatic digestion with collagenase type I 0.075% *w*/*v* (Worthington Biochemical Corporation, LakeWood, NJ, USA) for 30 min at 37 °C [43,44], filtration and centrifugation at 350× *g* for 4 min. The cell pellet obtained was suspended in complete medium, composed of Dulbecco’s Eagle Modified Medium (DMEM, Sigma-Aldrich, St. Louis, MO, USA) supplemented with 10% of Fetal Bovine Serum (FBS, GE Healthcare HyClone, Piscataway, NJ, USA) and 1% of Penicillin-Streptomycin-Glutamine (PSG, Thermo Fisher Scientific, Waltham, MA, USA) and then seeded at a density of 5000 cells/cm^2^.

Waste bone marrow samples were obtained from the femoral canal of male donors (58 ± 13 years old) who underwent total hip replacement. The bone marrow samples were rinsed in PBS and centrifuged for 10 min at 623× *g*. The mononuclear cells were plated at a density of 10,000 cells/cm^2^ in complete medium.

When 90% confluence was reached, both the hASCs and the human bone marrow-derived stem cells (hBMSCs) were detached by Trypsin-EDTA (Life Technologies, Carlsbad, CA, USA) 0,025% and then re-plated up to passage 4 when they were used for the following experiments.

### 2.3. Seeding Protocol and Experimental Set Up by DoE 

The set of experiments was performed on hASCs isolated from 3 different donors. LFAMs were first divided into portions of 10 mg and then rehydrated with complete medium. hASCs were added to LFAMs at a density of 15,000 cells/mg and maintained in an incubator at 37 °C and 5% CO_2_ for a maximum time of two h, based on the different times of adhesion designed by the DoE approach. The cell density (15,000 cells/mg) for a total of an amount of 10 mg LFAMs for each sample was considered as a fixed parameter and remained constant for all the experiments (Table 1). 

The standard protocol (SP) previously developed [36] consisting of two h of dynamic culture by an oscillating shaker (Rotamax 120, Heidolph) at 70 RPM, was considered as reference protocol and used as a starting point for the identification of the variable parameters (volume of cell/LFAMs suspension in the bioreactor, the dynamic culture modalities, the stirring speed and the duration of each protocol) (Table 1). 

The cell adhesion rate and the homogenous cell arrangement on the surface of LFAMs were considered the endpoints of the DoE analysis (Table 1). The DoE was performed by the JMP software (SAS Institute Inc.) that allows an optimized setup of experiments and a dependable and fast data analysis to be automatically obtained. After the setting up of the endpoints, the fixed and the variable input and process parameters, the statistical analysis permitted the definition of an optimized number of experiments (*n* = 13) to be performed. 

For each hASCs population, the 13 protocols were tested in triplicate. The dynamic culture of LFAMs/cells suspension was provided by a bioreactor system previously described [45]. Briefly, this bioreactor is a custom-made tube roller that permits a pre-settable dynamic culture to be obtained, as it is able to rotate at a programmable speed in continuous mode or with a defined pause between rotation cycles (Figure S1). 

Analyzing the outcomes of these 13 experiments, the DoE predicted an optimized final protocol (model) in terms of cell adhesion and cell arrangement on the surface of LFAMs (Table 2) that was then tested and validated. 

The validation step consisted of comparing the protocol predicted by the DoE with the other two reference seeding protocols, that are the standard protocol (SP: 70 RPM, 120 min, continuous, 1000 µL), already tested in the previous study [36], and the best protocol among the 13 tested. 

### 2.4. Evaluation of the Cell Adhesion Rate

For each protocol tested, the medium was removed to exclude non-adherent cells immediately after the end of the seeding phase. The total amount of adhered cells was evaluated by DNA quantification with the CyQuant assay (Invitrogen, Carlsbad, CA, USA) and an estimation of cell number based on the relative metabolic activity of each sample was obtained by Alamar Blue assay (Life Technologies, Carlsbad, CA, USA). Cell viability was calculated by metabolic activity normalized on the quantity of DNA in each sample. In detail, each LFAM/cells sample was incubated with a 10% *v*/*v* Alamar Blue solution for 4 h at 37 °C. Fluorescence was measured at Ex/Em 560/590 nm by a spectrophotometer (Victor X3, Perkin Elmer). The same samples were then harvested and lysed with Triton X-100 0.1% in ddH_2_O for the DNA content evaluation by CyQuant cell proliferation Assay Kit. Fluorescence was read at 520 nm (excitation 480 nm).

Evaluation of cell adhesion was performed with Calcein staining (Life Technologies): each sample was treated with 2 µM of Calcein-AM in saline solution for 10 min at 37 °C and 5% CO_2_. The auto-fluorescence of silk fibroin after exposure to green light was used to better discriminate the surface of adhesion [46]. Then, micrographs were obtained by observing cells with a fluorescence microscope (Olympus IX71). For each experimental condition, a quantification of the adherent Calcein-stained cells per single LFAMs was performed by ImageJ software. Briefly, three representative images for each experimental condition were selected and then used for the semi-quantitative analysis. The threshold level was modified in order to discriminate green fluorescent cells and the “Analyze Particles” command was used for the cell count; particles with a size less than 10 pixel^2^ were ignored.

### 2.5. Statistical Analysis

DoE was performed using JMP (SAS Institute software). A DoE custom design was generated for the study, defining cell adhesion rate and cell arrangement on the surface of LFAMs, obtained from the quantification of DNA of adhered cells and the assessment of their metabolic activity, as outcomes to be maximized. The time (min), the stirring speed (RPM), the dynamic culture modalities (intermittent or continuous) and the volume of LFAMs/cells suspension (µL) were defined as variable process parameters. The software automatically generates the design of the experiments to perform. After the experiments, the obtained data were inserted in the software and the screening effect was evaluated for each single output and for the overall results. The personality of the model was set at standard least squares whereas the emphasis set to effect screening.

Statistical analyses of data were performed by GraphPad Prism v5.0 software (GraphPad Software Inc., La Jolla, CA, USA). Data are expressed as the mean ± standard deviation (SD). The values distribution was assayed by the Kolmogorov–Smirnov normality test. For normally distributed data, the student T-test or the one-way analysis of variance (ANOVA) were performed to compare groups. If the data were not normally distributed, the Mann–Whitney test or Kruskal–Wallis test were applied: *p* values < 0.05 were considered statistically significant. 

## 3. Results

### 3.1. LFAMs Properties

**Figure 1:**
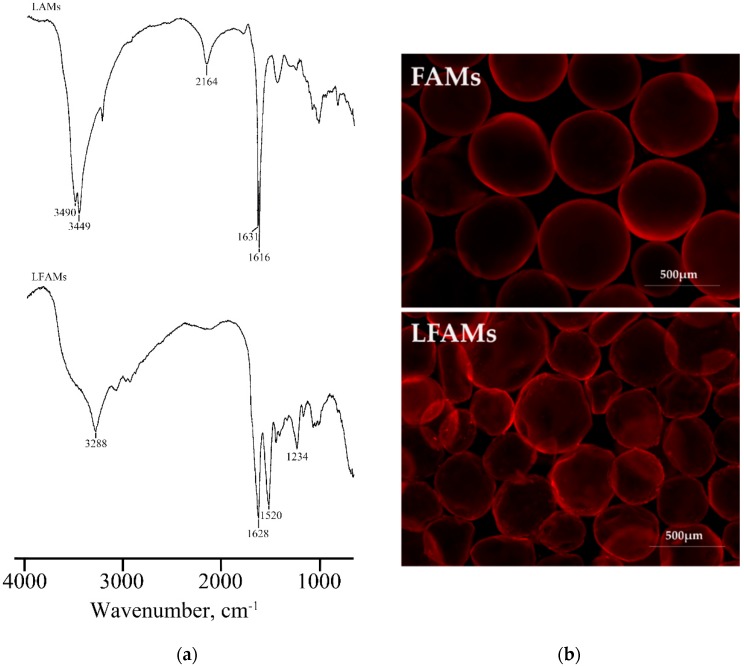
Fourier transform infrared (FT-IR) spectra of lyophilized alginate microcarriers (LAMs) and lyophilized fibroin-coated alginate microcarriers (LFAMs) in the spectral region 4000–1000 cm^−1^ (**a**). Representative micrographs of fibroin-coated microcarriers (FAMs) and LFAMs after exposition at green light. Magnification 4X (**b**).

Size distribution of FAMs and LFAMs were evaluated using a light scattering granulometer; LFAMs were previously rehydrated, with water at 37 °C for 2 h in ddH_2_O, mimicking the hydration procedure used before the cell adhesion tests. Our results demonstrated that, after the rehydration, LFAMs showed the same particle size distribution with respect to FAMs (462.97 ± 160.25 µm and 418.14 ± 59.58 µm, respectively). These results demonstrated that the freeze-drying process did not affect the microcarrier structure and it can be considered an effective strategy to obtain a dry, stable, and ready-to-use product. FT-IR analysis was performed on both LAMs and LFAMs to evaluate the fibroin and alginate molecular conformation (Figure 1a). 

The FT-IR spectra of LAMs demonstrated the presence of calcium alginate absorption bands; in particular, in the range 3000–3600 cm^−1^ the bands were related to the stretching vibration of O–H bonds, while the ones at 1616 and 1631 cm^−1^ were correlated to the asymmetric stretching vibration of carboxylate group. On the other side, the FT-IR spectra of LFAMs showed the typical absorption bands of fibroin used for the coating of alginate microcarriers. The presence of two bands at ~1620 and 1520 cm^−1^ demonstrated the stable β-sheet conformation of silk fibroin coating as previously demonstrated by other authors [47,48]. The band detected at 1628 cm^−1^ was ascribed to the C=O stretching of Amide I groups, while at 1520 cm^−1^ the C–N stretching and the N–H blending of Amide II were detected. The presence of fibroin coating was also confirmed by the absorption band at 3288 cm^−1^, observable only in the LFAMs spectra; this peak was attributable to the N–H stretches of fibroin amines.

The auto-fluorescence of silk fibroin after exposure to green light was exploited to morphologically observe microcarriers, before (FAMs) and after (LFAMs) the lyophilization process. In general, when lyophilized, the microcarrier structure is well maintained and it does not show any kind of damage. The rounded shape of microcarriers is preserved even if LFAMs present some small deformation with respect to the FAMs (Figure 1b). Furthermore, the stained surface of LFAMs (Figure 1b) demonstrated that lyophilization and rehydration did not induce any structural and morphological modification, in terms of shape and fibroin coating integrity.

### 3.2. Quantification of Cell Adhesion Rate on the LFAMs Surface

**Figure 2:**
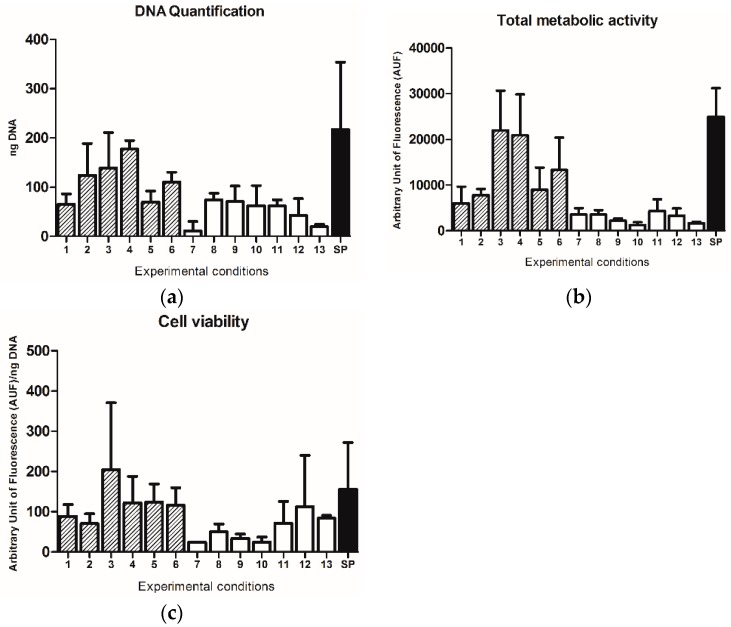
(**a**) DNA quantification, (**b**) total metabolic activity and (**c**) cell viability of samples for each protocol (1: 20 RPM, 30 min, intermittent, 1000 µL; 2: 20 RPM, 120 min, intermittent, 1000 µL; 3: 10 RPM, 60 min, intermittent, 400 µL; 4: 10 RPM, 120 min, intermittent, 400 µL; 5: 5 RPM, 30 min, intermittent, 400 µL; 6: 5 RPM, 90 min, intermittent, 1000 µL; 7: 20 RPM, 60 min, continuous, 400 µL; 8: 20 RPM, 90 min, continuous, 400 µL; 9: 10 RPM, 30 min, continuous, 1000 µL; 10: 10 RPM, 60 min, continuous, 1000 µL; 11: 10 RPM, 120 min, continuous, 400 µL; 12: 5 RPM, 30 min, continuous, 400 µL; 13: 5 RPM, 120 min, continuous, 1000 µL; SP: 70 RPM, 120 min, continuous, 1000 µL). Results are reported as mean ± SD.

Although no statistically significant differences were observed, higher DNA content was found in samples seeded with intermittent dynamic protocols (10 min of static and 10 min of dynamic cell culture; protocols 1–6), in comparison with those seeded under a continuous dynamic condition (protocols 7–13) (Figure 2a). 

The amount of cell adhesion was also assessed by the detection of total metabolic activity by Alamar Blue assay. The highest values were obtained under protocol 3 (3: 10 RPM, 60 min, intermittent, 400 µL) and 4 (4: 10 RPM, 120 min, intermittent, 400 µL) (Figure 2b). This observation suggests that independently of the time of seeding, the use of the intermittent dynamic culture at a speed of 10 RPM in a volume of LFAMs/cells suspension of 400 µL may represent the best combination of factors to promote cell adhesion. 

No relevant differences were observed by comparing all the protocols in terms of cell viability, defined as metabolic activity normalized on DNA content of each sample, as shown in Figure 2c, thus suggesting that no significant effects on cell viability were determined by all the different experimental set-ups.

Observing the data obtained using the standard protocol, higher levels of DNA content (Figure 2a), metabolic activity (Figure 2b), and cell viability (Figure 2c), were found with respect to all the other protocols tested.

### 3.3. Qualitative Evaluation of Homogeneity in Cell Adhesion

**Figure 3:**
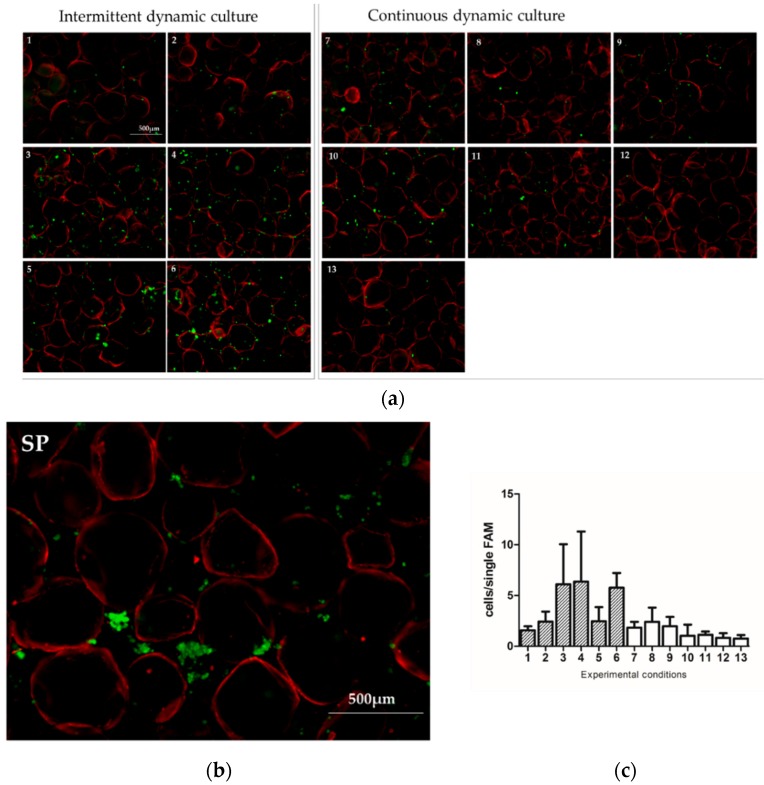
Representative images of Calcein stained cells (green) seeded on LFAMs (auto-fluorescent in red) with the 13 different protocols suggested by Design of Experiment (DoE) (**a**) (1: 20 RPM, 30 min, intermittent, 1000 µL; 2: 20 RPM, 120 min, intermittent, 1000 µL; 3: 10 RPM, 60 min, intermittent, 400 µL; 4: 10 RPM, 120 min, intermittent, 400 µL; 5: 5 RPM, 30 min, intermittent, 400 µL; 6: 5 RPM, 90 min, intermittent, 1000 µL; 7: 20 RPM, 60 min, continuous, 400 µL; 8: 20 RPM, 90 min, continuous, 400 µL; 9: 10 RPM, 30 min, continuous, 1000 µL; 10: 10 RPM, 60 min, continuous, 1000 µL; 11: 10 RPM, 120 min, continuous, 400 µL; 12: 5 RPM, 30 min, continuous, 400 µL; 13: 5 RPM, 120 min, continuous, 1000 µL) and the Standard Protocol (**b**) (SP: 70 RPM, 120 min, continuous, 1000 µL). Scale bar = 500 µm. Semi-quantitative evaluation of Calcein-stained cells on the surface of LFAMs (**c**).

To assess the cell arrangement on the surface of LFAMs, Calcein staining was performed immediately after the end of the seeding phase (Figure 3). 

The use of the standard protocol induced the formation of cell clusters instead of a homogeneous cell arrangement on LFAMs. Again, the highest adhesion rate and the most homogenous cell adhesion were obtained when an intermittent dynamic culture was used (Figure 3a, protocols 1–6), especially using protocols 3, 4, and 6. This evidence was confirmed by the semi-quantitative analyses of sample that revealed a higher amount of Calcein-stained cells adherent on the surface of the LFAMs (Figure 3c). Well-distributed adherent cells were found on the surface of LFAMs in the samples seeded with the intermittent dynamic conditions, in contrast with what was observed in samples seeded with the standard protocol. 

### 3.4. Influence of Single Parameters on the Cell Adhesion Rate

**Figure 4:**
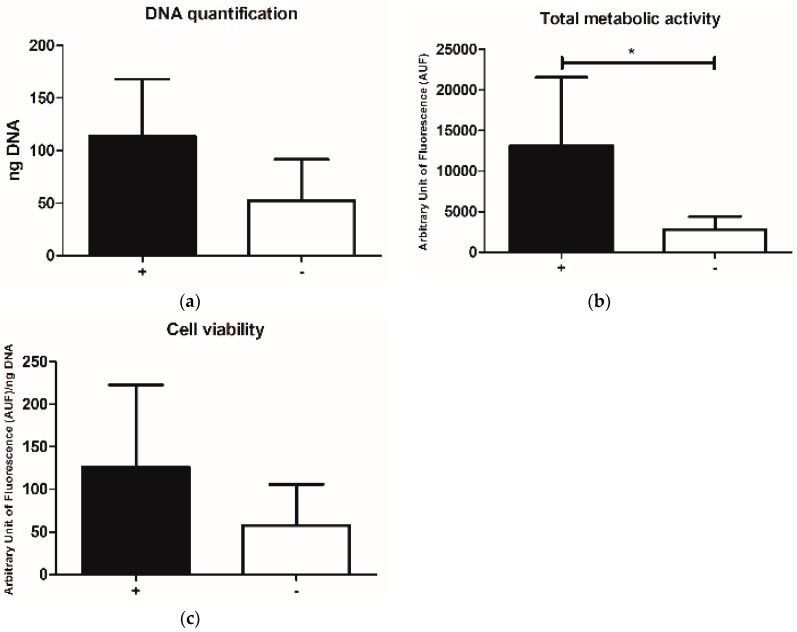
(**a**) DNA quantification, (**b**) total metabolic activity and (**c**) cell viability of samples grouped for the intermittent (+) vs. continuous (−) dynamic culture. Results are reported as mean ± SD. * *p* < 0.05.

Grouping all data for intermittent vs. continuous dynamic culture, the estimated number of cells, meant as both DNA content and total metabolic activity was higher in the intermittent group, in a significant manner, at least for that concerning total metabolic activity (*p* < 0.05) (Figure 4a,b).

Similarly, cell viability resulted in being slightly affected, showing a higher level in samples cultured with the intermittent dynamic protocols, even if no statistically significant differences were observed (Figure 4c).

**Figure 5:**
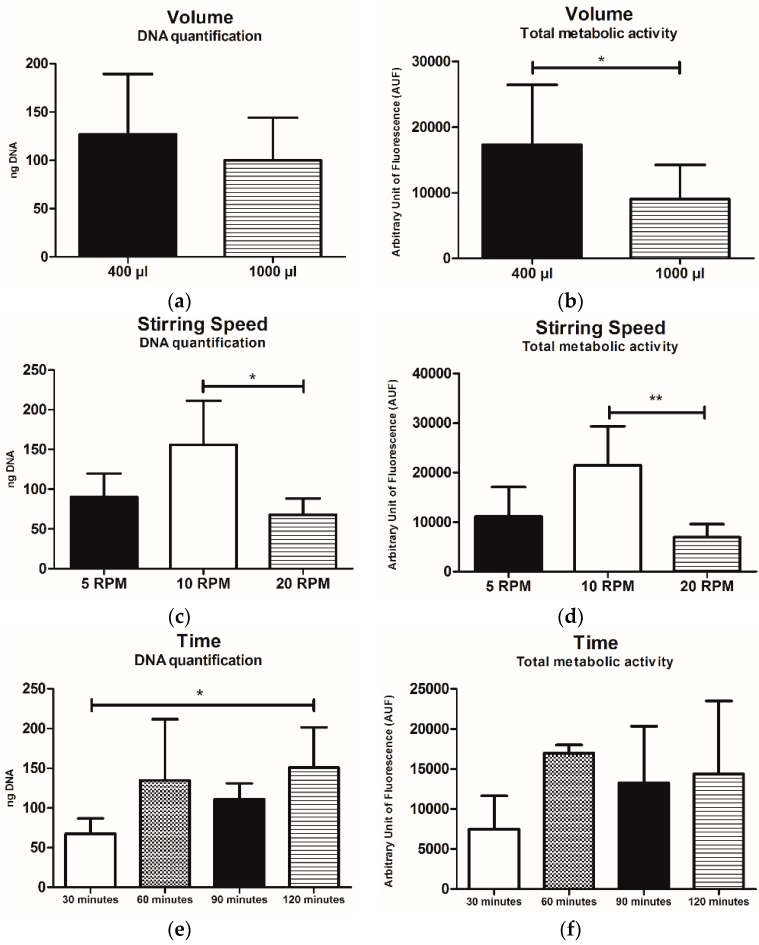
(**a**,**c**,**e**) represent the DNA content while (**b**,**d**,**f**) represent the total metabolic activity of samples cultured with intermittent dynamic protocols (1–6), grouped for volume LFAMs/cells suspension (µL) (**a**,**b**), stirring speed (RPM) (**c**,**d**) and time of the process (min) (**e**,**f**). Data are expressed as mean values ± SD. * *p* < 0.05, ** *p* < 0.01.

Focusing on the data derived from intermittent dynamic protocols only, the samples where a small volume of LFAMs/cells suspension (400 µL) was used showed higher values in terms of DNA content and total metabolic activity (*p* < 0.05) with respect to those with the larger one (1000 µL) (Figure 5a,b). 

The seeding speed of 10 RPM allowed for the obtainment of the highest values with respect to samples seeded at 5 RPM and 20 RPM (Figure 5c,d), in term of both DNA content and total metabolic activity. For what concerns DNA content, this difference resulted in being statistically significant (* *p* < 0.05 and ** *p* < 0.01, vs. 5 RPM and 20 RPM, respectively) (Figure 5c,d). 

Regarding the time of process, 120 min was the time able to guarantee the highest DNA amount in respect to the other conditions (30 min, 60 min, and 90 min) with a statistically significant difference (*p* > 0.05) when compared with the 30 min ones (Figure 5e). No relevant differences were observed in terms of total metabolic activity even if the highest value was obtained in the samples cultured for 60 min (Figure 5f).

### 3.5. Identification of the Optimal Experimental Condition to Optimize the Cell Adhesion Process onto LFAMs 

**Figure 6:**
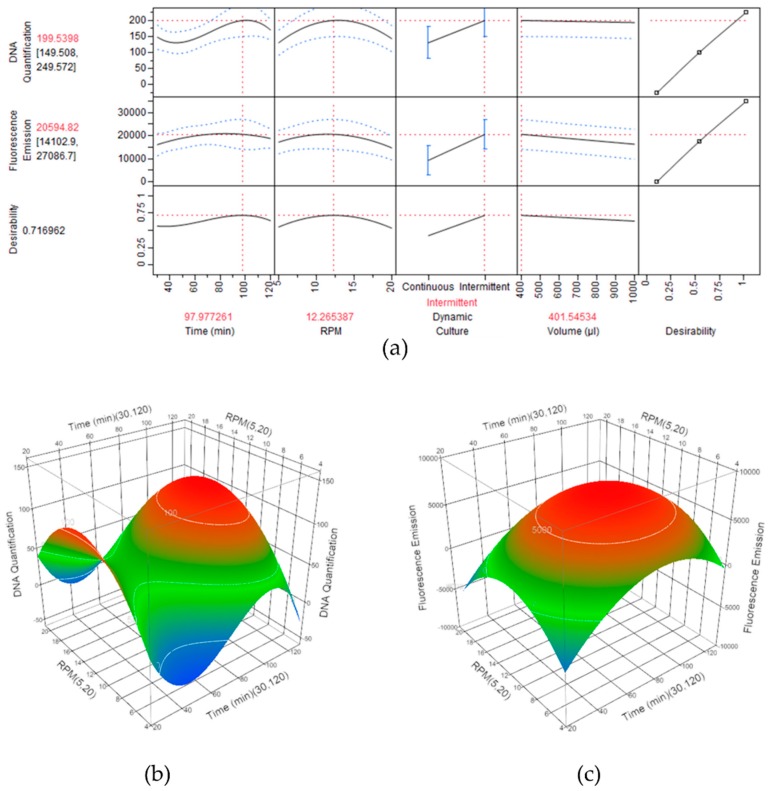
DoE outcome reporting the optimal parameter configuration obtained maximizing the desirability in the statistical software. (**a**) Extraction of the optimal combination by the maximization of the input variables. (**b**) Surface profiles of the DNA quantification and (**c**) of the total metabolic activity, related to the seeding time (min) and the stirring speed (RPM). (red: maximum efficiency; green: medium efficiency; blue: low efficiency).

The DoE approach allowed for a complete screening of the effects on the cell seeding derived from the modification of the variable process parameters. A prediction profile was generated by the JMP software, showing the synergistic effect and the relative desirability values of each parameter in relation to the final output. The optimal experimental parameters combination resulting from the DoE analysis was obtained maximizing the desirability, corresponding to an intermittent dynamic culture with a duration of 98 min, at a speed of 12.27 RPM, in a seeding volume of 401.5 µL, having the highest overall desirability score (0.717) (namely model protocol) (Figure 6a). 

Considering the intermittent dynamic protocols only and the effect of the seeding time versus the stirring speed (RPM), two surface profiles were generated for DNA content and metabolic activity. Theoretically, the maximum DNA content would be obtained with 100 min of seeding time at 13 RPM (Figure 6b), while the highest total metabolic activity value would result when using 84 min of seeding time at 11.5 RPM (Figure 6c).

### 3.6. Validation of the Optimized Protocol

To validate the ability of the protocol theoretically predicted by DoE (model: 12.27 RPM, 98 min, intermittent, 401.5 µL) to outperform the standard protocol (SP: 70 RPM, 120 min, continuous, 1000 µL), and the best performing protocol tested (3: 10 RPM, 60 min, intermittent, 400 µL), three populations of hASC and three of hBMSCs were seeded on LFAMs under these conditions.

**Figure 7:**
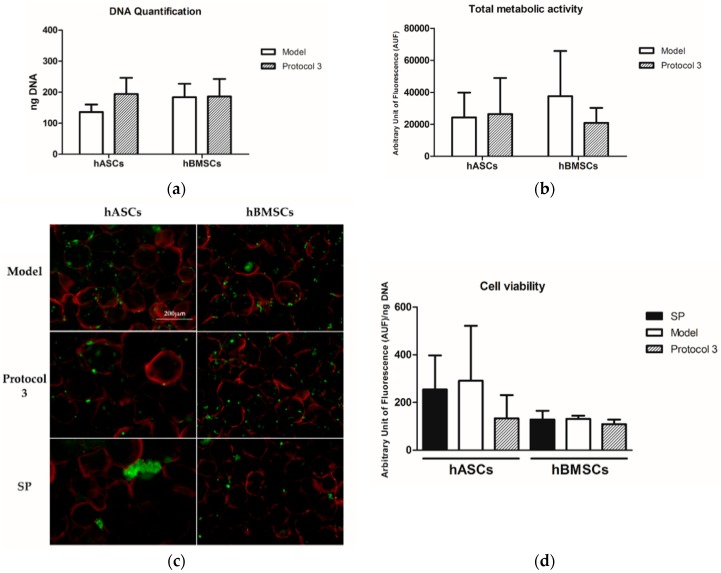
(**a**) DNA quantification and (**b**) total metabolic activity of cells cultured with the DoE-predicted model (**model**: 12.27 RPM, 98 min, intermittent, 401.5 µL) and with protocol 3 (**3**: 10 RPM, 60 min, intermittent, 400 µL). Results are reported as mean ± SD. (**c**) Representative images of Calcein stained cells seeded on LFAMs with the DoE-predicted model, Protocol 3 and the Standard Protocol (**SP**: 70 RPM, 120 min, continuous, 1000 µL). Scale bar = 200 µm. (**d**) Viability of hASCs and hBMSCs after being cultured with the same protocols.

When the model protocol was applied, the DNA amount of adhered cells on LFAMs was 136 ± 24 ng and 183 ± 44 ng for hASCs and hBMSCs, respectively (Figure 7a). 

These values were similar to those obtained with protocol 3 (194 ± 52 ng for hASCs and 186 ± 56 ng for hBMSCs). 

The total metabolic activities of hASCs detected after seeding with the model protocol and protocol 3 were almost the same whereas slight differences were observed in hBMSCs, where the model protocol showed higher values (Figure 7b). Again, the standard protocol allowed for higher DNA content and metabolic activity, but these data were affected by the presence of cell aggregates rather than LFAM-adherent cells. 

Indeed, Calcein staining confirmed that the standard protocol led to non-homogenous cell adhesion for both hASCs and hBMSCs, with clear cell aggregates around the LFAMs, as already shown in the previous study (Figure 7c). On the contrary, a homogenous cell adhesion on the surface of LFAMs was observed in samples seeded with the model protocol and protocol 3, without significant difference between them. The cell viabilities of both hASCs and hBMSCs were similar irrespective of the seeding protocols applied (Figure 7d).

## 4. Discussion

The main result of this methodological study is the identification of the optimal seeding condition to obtain MSCs adhesion on FAMs in a timeframe compatible with one-step clinical procedures, allowing for the maintenance of cell viability and associated to homogeneous cell distribution. In our previous works, we demonstrated that FAMs possess all the features of being a reliable cell delivery system for MSCs [36,37]. In this study, different seeding protocols were identified by DoE to test the homogeneity of cell distribution while keeping a high adhesion rate on the surface of the carriers. 

Fibroin-coated microcarriers were selected as a delivery system since they had been widely used in several applications, especially when cell growth was desirable [49,50,51,52]. Indeed, the main interest in the use of microcarriers for cell culture was driven by the optimal surface–area/volume ratio that represents a great advantage in term of cost-effectiveness, reducing the amount of the materials and the time needed for monolayer cell expansion [53]. Moreover, the use of fibroin as a support for cell growth was considered a suitable approach for MSCs culture to provide not only a good cellular expansion but also a good maintenance of cell proliferation and differentiation capability [54,55,56,57,58]. Finally, microcarriers were defined as a suitable cell delivery system since they can be directly injected locally, ensuring the maintenance of cells at the target site while minimizing patients’ discomfort [25,59,60]. 

In our case, the development of FAMs was thought to facilitate and improve the local injective delivery of MSCs for musculoskeletal applications. Indeed, one-step procedures represent a convenient and cost-effective approach to exploit the MSCs potential for the treatment of many conditions, including osteoarthritis, the most common form of joint arthritis. However, although many studies have demonstrated the feasibility and effectiveness of the use of MSCs in the treatment of these pathologies, the technical aspects related to the cell delivery could be improved to enhance clinical translation. In particular, given the lower number of MSCs obtained intraoperatively from bone marrow or adipose tissue, the effectiveness of the local delivery of progenitor cells and their permanence at the injury site is crucial to achieve therapeutic results. Moreover, the possibility to deliver MSCs while adhered to a surface rather than in a liquid suspension would allow a more physiological cell environment to be maintained, eventually resulting in a more prompt and increased activity of MSCs, especially in terms of paracrine action [24]. While other approaches for one-step applications, such as the direct injection in the target tissue of cells in suspension or the delivery of cells embedded in capsules or hydrogels might allow for more rapid cell administration [61,62], they prevent the maintenance of this peculiar feature of MSCs, likely reducing the safety and efficacy of the system [63]. 

With respect to our previous study describing the feasibility of this approach [36], in this work a more stable formulation of FAMs, in the form of a lyophilized product (LFAMs) was tested. The shelf life of freeze-dried microcarriers (LFAMs) was higher than FAMs; the lyophilization process represents an effective strategy to reduce the water content and to improve the stability of developed microcarriers. The obtained results demonstrated that LFAMs maintained all their features in terms of physico-chemical, size distribution, and cell adhesion properties with respect to FAMs, which were analyzed in our previous work [36]. In particular, by rehydrating LFAMs for 2 h, it was possible to obtain the same size distribution of fresh fibroin-coated microcarriers (FAMs). Furthermore, FT-IR analysis demonstrated that after lyophilization the silk fibroin coating maintained its stable conformation. The lyophilization process allowed us to obtain a dry and more stable product with respect to fresh microcarriers; for these reasons the freeze-drying technique could be considered a good strategy to improve the technological process of FAMs production, ameliorating the preservation and the reproducibility of the process and opening the perspective for a possible future off-the-shelf use of LFAMs.

As the main aim of this study was to develop a protocol of use of LFAMs compliant with the minimal requirements for MSCs delivery in a one-step clinical application, a low cell number (15,000 cells/mg of LFAMs) and a short time for the adhesion of cells (maximum 2 h) were applied to mimic as closely as possible the clinical setting. 

To improve the previously proposed seeding protocol, the parameters that might influence the seeding efficiency were tested in multiple combinations using the DoE approach. 

Our results indicated that hASCs were able to adhere to LFAMs in less than 2 h, under suitable conditions. In particular, the dynamic seeding of cells provided the best outcomes in comparison with static cultures, in terms of cell adhesion and viability, confirming previously published literature findings [64,65]. Moreover, the modality of the dynamic culture—intermittent or not—deeply influences final cell adhesion and subsequent expansion and differentiation. Indeed, our results showed that the intermittence was the most influencing parameter among those tested in this study, thus confirming the hypothesis that the intermittent dynamic culture is able to improve the adhesion rate [66]. However, the formation of cell aggregates, particularly frequent in continuous dynamic culture, as well as cell damage and detachment from microcarriers should be prevented. The protocols consisting of intermittent dynamic seeding provided the most homogenous cell adhesion of FAMs, without formation of cell aggregates even if the lowest hydrodynamic shear stress to the cells has to be guaranteed to avoid cell damage or detachment [67,68,69]. 

The main limitation of this work is the use of cultured and expanded cells, while the one-step procedure would require freshly isolated cells, in the form of bone marrow aspirate concentrates (BMAC) or stromal vascular fraction (SVF). The rationale of using expanded cells resides in the methodological nature of the present paper, requiring a high number of cells in order to perform all the experiments and the related analysis. Further experiments are needed to confirm the effectiveness of the protocol developed in the present study for the intra-operative administration of injectable products such as SVF and BMAC, in the view of the application of this combined approach at the point of care.

## 5. Conclusions

This study shows significant insights on the use of LFAMs as a cell delivery system with a perspective clinical relevance. LFAMs support rapid hASCs adhesion and good maintenance of their viability. In addition, the optimized protocol suggested by the DoE analysis also permits a homogenous arrangement of cells on the surface of the carrier. The lyophilization technique allowed us to obtain a dry, stable, and ready-to-use product, which maintained the original properties of the fibroin-coated microcarriers. Overall, LFAMs and the protocol of use proposed in this study comply with the minimal requirements for the assessment of MSCs delivery in a one-step clinical application.

## Figures and Tables

**Table 1:** Endpoints of the study. Setting up of fixed and variable parameters. LFAMs = lyophilized fibroin-coated alginate microcarriers.

**Endpoints**	Cell Adhesion Rate Cell Arrangement	
**Fixed process parameters**	Cell density (15,000 cells/mg) LFAMs/sample (10 mg)	
**Variable process parameters**	Length of time	30 min
		60 min
		90 min
		120 min
	Stirring speed	5 RPM
		10 RPM
		20 RPM
	Dynamic culture	Intermittent
		Continuous
	Volume of LFAMs/cells suspension (3.75 × 10^5^ cells and 10 mg of LFAMs)	400 µL
		1000 µL

**Table 2:** Design of Experiment (DoE)-selected protocols resulting by combination of the variable parameters.

	Variable Parameters			
Protocol	Stirring Speed (RPM)	Time (min)	Dynamic Culture Intermittent (+) or Continuous (−)	Volume (µL)
1	20	30	+	1000
2	20	120	+	1000
3	10	60	+	400
4	10	120	+	400
5	5	30	+	400
6	5	90	+	1000
7	20	60	−	400
8	20	90	−	400
9	10	30	−	1000
10	10	60	−	1000
11	10	120	−	400
12	5	30	−	400
13	5	120	−	1000

## Supplementary Materials

The following are available online at http://www.mdpi.com/1999-4923/10/4/200/s1, Figure S1: Image of the pre-settable bioreactor used. Aliquots of LFAMs/cells suspension were maintained in intermittent or continuous dynamic conditions for the duration of each seeding protocol.
