# Supplementary material for: Silk/Fibroin Microcarriers for Mesenchymal Stem Cell Delivery: Optimization of Cell Seeding by the Design of Experiment
Source: Pharmaceutics. 2018 Oct 24;10(4):200. doi: 10.3390/pharmaceutics10040200 (PMC6321597; doi:10.3390/pharmaceutics10040200)
Supplement: Supplementary file 1 [file pharmaceutics-10-00200-s001.pdf]

pharmaceutics

Supplementary Materials: Silk/Fibroin Microcarriers for Mesenchymal Stem Cell Delivery: Optimization of Cell Seeding by the Design of Experiment

Carlotta Perucca Orfei, Giuseppe Talò, Marco Viganò, Sara Perteghella, Gaia Lugano, Francesca Fabro Fontana, Enrico Ragni, Alessandra Colombini, Paola De Luca, Matteo Moretti, Maria Luisa Torre and Laura de Girolamo

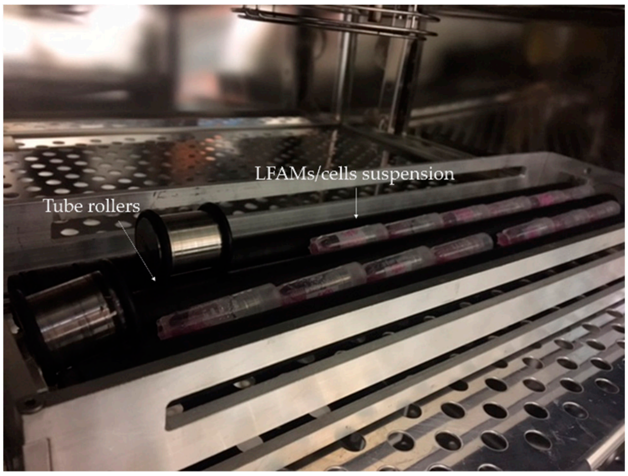

Figure S1. Image of the pre-settable bioreactor used. Aliquots of lyophilized fibroin-coated alginate microcarriers (LFAMs)/cells suspension were maintained at intermittent or continuous dynamic conditions for the duration of each seeding protocol.
